# Melatonin attenuates chronic sleep deprivation‐induced cognitive deficits and HDAC3‐Bmal1/clock interruption

**DOI:** 10.1111/cns.14474

**Published:** 2023-09-18

**Authors:** Yujie Hu, Yefan Lv, Xiaoyan Long, Guoshuai Yang, Jinxia Zhou

**Affiliations:** ^1^ Department of Neurology, Xiangya Hospital Central South University Changsha China; ^2^ Department of Neurology Haikou Affiliated Hospital of Central South University Xiangya School of Medicine Haikou China

**Keywords:** Bmal1, circadian rhythm, clock, cognition, HDAC3, melatonin, sleep deprivation

## Abstract

**Background and Aims:**

Sleep is predicted as a key modulator of cognition, but the underlying mechanisms are poorly understood. In this study, we investigated the effects of melatonin on chronic rapid eye movement sleep deprivation (CRSD)‐induced cognitive impairment and circadian dysfunction in rat models.

**Methods:**

Thirty‐six Sprague‐Dawley male rats were divided into three groups: CRSD with saline treatment, CRSD with chronic melatonin injection (20 mg/kg/day), and non‐sleep‐deprived control. The cognitive behavioral tests as well as the expression of clocks and HDAC3 were evaluated in all groups.

**Results:**

CRSD significantly reduced recognition index in novel object location, increased escape latency and distance traveling in Morris water maze while melatonin treatment attenuated CRSD‐induced hippocampal‐dependent spatial learning and memory deficits. Furthermore, the mRNAs of *brain and muscle aryl hydrocarbon receptor nuclear translocator‐like 1(Bmal1)* and *circadian locomotor output cycles kaput* (*Clock*) were globally down‐regulated by CRSD with constant intrinsic oscillation in both hippocampus and peripheral blood. The protein levels of hippocampal Bmal1, Clock, and HDAC3 were also remarkably down‐regulated following CRSD. Melatonin treatment reversed CRSD‐induced alterations of Bmal1/Clock and HDAC3 on both mRNA levels and protein levels.

**Conclusions:**

Our data indicate that melatonin treatment attenuates CRSD‐induced cognitive impairment via regulating HDAC3‐Bmal1/Clock interaction. These findings explore a broader understanding of the relationship between sleep and cognition and provide a potential new therapeutic target for cognitive impairment.

## Graphical abstract

Melatonin treatment attenuates chronic rapid eye movement sleep deprivation‐induced hippocampal‐dependent spatial learning and memory deficits via regulating HDAC3‐Bmal1/Clock interaction.

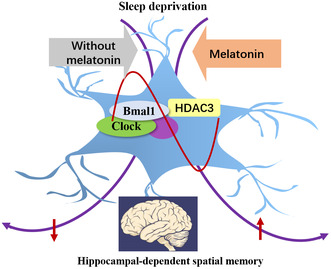

## 1 INTRODUCTION

Although humans spend 30% time of their life on sleep, the physiological function of sleep remains unclear. Sleep consists of two alternating stages: non‐rapid eye movement (NREM) sleep and rapid eye movement (REM) sleep. It plays a critical, but still poorly understood role in brain homeostasis and the regulation of neural physiology. In consequence, inadequate sleep and sleep deprivation impair cognitive function and health, promoting the development of neuropsychiatric, metabolic, and immunologic disorders.^1^

The sleep–wake cycle refers to the biological pattern of alternating sleep and wakefulness with a periodicity approximating the 24 h day–night cycle.^2^ It is governed by two major processes: a circadian process (also known as process C) and a homeostatic process (process S).^3^ The circadian rhythm is a distributed process that brain regions and multiple peripheral organ systems exhibit inherent oscillatory capacity. The phasing and amplitude of these distributed cell populations are set by the suprachiasmatic nucleus (SCN) of the hypothalamus.^4^ SCN neurons generate rhythmic electrical activity and produce synchronizing signals that control the phases of the oscillations of so‐called peripheral clocks operating in other tissues.^5^ The mechanisms underlying circadian rhythms involve circadian oscillations in gene expression, protein modifications, and hormone secretion. These oscillations are controlled by the products of core circadian clock genes built from a delayed negative transcriptional feedback loop. The brain and muscle aryl hydrocarbon receptor nuclear translocator‐like 1(Bmal1) and circadian locomotor output cycles kaput (Clock) proteins act as the major transcriptional activators while the Period (Per1, Per2, Per3) and Cryptochrome (Cry1, Cry2) proteins act as the major repressors.^6^ The homeostatic process, which is the need for sleep (sleep pressure) as a function of the time since the last adequate sleep, is an indicator of homeostatic sleep drive. These two processes interact to determine the timing of sleep onset and offset, as well as the stability of waking neurocognitive functions.^3^, ^7^, ^8^

Melatonin is an amphiphilic neurohormone synthesized and secreted by the pineal gland mainly during the dark period of the circadian cycle, promoting sleep, and synchronizing central and peripheral rhythms.^9^ Endogenous melatonin is widely used alone or in combination with core body temperature as a phase marker of the circadian pacemaker. The secretion of melatonin is regulated by the expression of clock genes in the SCN region. Conversely, melatonin provides feedback to the SCN clock, causing the mutual conversion of circadian rhythms and maintaining the normal sleep cycle.^10^ Clinical trials have demonstrated that exogenous melatonin is effective for treating several types of sleep disorders, including circadian rhythm sleep–wake disorder, insomnia, and parasomnia.^4^, ^11^ However, the effects of melatonin on cognition are still under debate. An early study on healthy participants presents no association between endogenous melatonin levels and cognitive function.^12^ But later studies show a significant association between physiological melatonin levels and cognition in a large cross‐sectional study on the general elderly population^13^ as well as in healthy young adults.^14^ Furthermore, administration of exogenous melatonin is reported to attenuate cognitive impairment in animal models^15^, ^16^, ^17^, ^18^ and human^19^, ^20^ while these protective effects for cognition are not observed in other studies.^21^, ^22^, ^23^, ^24^, ^25^

Histone acetylation is modulated by the activity of histone acetyltransferase (HAT) and histone deacetylase (HDAC) and plays an important role in the regulation of clock genes and cognition.^9^, ^26^ HDAC3, the most highly expressed class I HDAC in the brain, has been shown to mediate circadian clock output pathways^26^, ^27^ and regulate learning and memory.^26^, ^28^

Given the regulatory role of melatonin on circadian rhythms and sleep, we proposed it could be protective for specific sleep interruption‐related cognitive impairment. Furthermore, we wished to explore the common mechanistic pathways in the regulation of clock biology and cognition, in particular the role of HDAC3. In this study, we investigated the effects of melatonin on chronic REM sleep deprivation (CRSD)‐induced cognitive impairment and circadian dysfunction in rat models. We show that melatonin attenuates CRSD‐induced hippocampal‐dependent spatial learning and memory impairment and reverses the alterations of HDAC3 and circadian rhythms in rats.

## 2 MATERIALS AND METHODS

### 2.1 Experiment design

**FIGURE 1:**
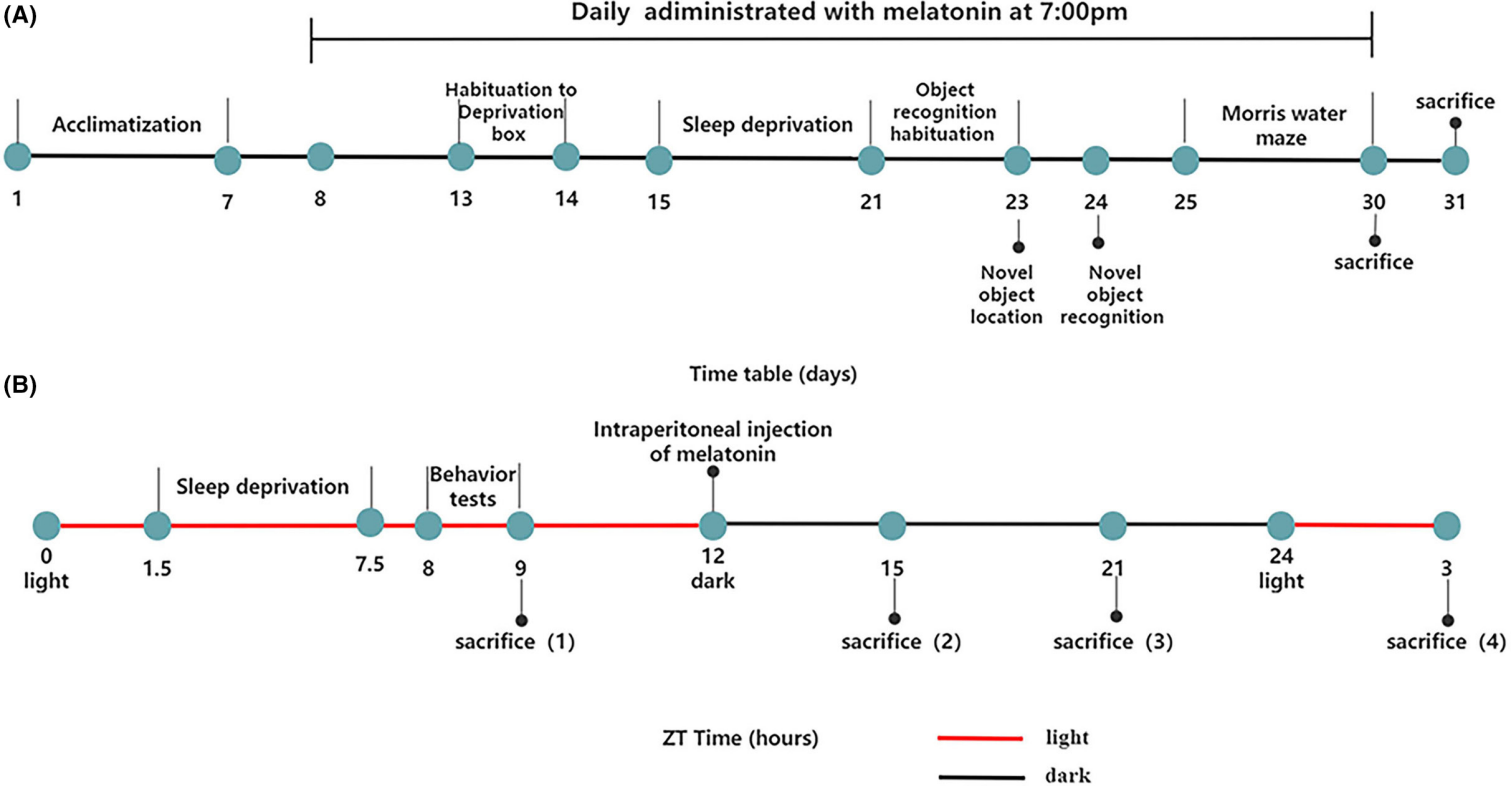
Schematic representation of the experiment protocol on days (A) and zeitgeber time (B). (A) Rats were acclimatized from Days 1 to 7 followed by intraperitoneal 20 mg/kg/day melatonin or the same volume of saline injection for a total of 23 days. Sleep deprivation was conducted on the 15th day for seven consecutive days. After sleep deprivation, rats were habituated to an arena for 10 min each day for consecutive 3 days (Days 21–23) followed by novel object location tests (Day 23), novel object recognition tests (Day 24), and Morris water maze (days 25–30). All rats were sacrificed on Days 30 and 31 at different time points. (B) On zeitgeber time (ZT), ZT0 (7:00) is set as the light‐on time and ZT12 (19:00) is set as the light‐off time. The red line represents day and the black line represents night. Sleep deprivation was conducted from ZT1.5 to ZT7.5. All behavior tests were conducted at ZT8 to ZT9 and drug injections were administrated at ZT12. At the end of the experiments, rats were sacrificed at ZT9 (Day 30), ZT15 (Day 30), ZT 21 (Day 30), and ZT3 (Day 31), respectively.

All experimental procedures were performed under the Institutional Guidelines of Care and Use of Animals and approved by the Animal Ethics Committee of Central South University of China (no. 2017‐S176). Eight‐week‐old Sprague‐Dawley (SD) male rats (*n* = 36, weighing 200–220 g) were purchased from the Laboratory Animal Center of Central South University (Changsha, China). Rats were housed in 16 cages (5–6 rats/cage) in a climate‐controlled room (23–25°C, 40%–60% humidity) with a regular 12/12‐h light/dark cycle. For tests performed on animals maintained under this light cycle, we used the zeitgeber time (ZT) nomenclature, with ZT0 (7:00) set as the light‐on time, and ZT12 (19:00) set as the time for light‐off. All rats had free access to food and water. After adaption for 1 week, 36 rats were randomly divided into three groups: CRSD with saline treatment group (CRSD + NS, *n* = 12), CRSD with melatonin treatment group (CRSD + MT, *n* = 12), and non‐sleep‐deprived control group (CON + NS, *n* = 12). Melatonin (M5250; Sigma) was dissolved in 20% ethanol in saline.^29^ From Day 8, rats in the CRSD + MT group were intraperitoneally injected with a single dose of 20 mg/kg/day at ZT12 (19:00) for a total of 23 days.^29^, ^30^, ^31^ The other two groups (CRSD + NS, CON + NS) of rats received the same volume of saline intraperitoneal injection with a coordinated content of ethanol at the same time. Sleep deprivation was conducted from Days 15 to 21 followed by behavior tests. Rats were sacrificed and tissues were harvested at different time points on Day 30 (ZT9, ZT15, ZT21) and Day 31 (ZT3) after all behavior tests were completed. The general timetables of the experiment design were presented in Figure 1.

### 2.2 Chronic REM sleep deprivation

CRSD was induced using multiple columns surrounded by water (modified multiple platform model) as previously reported.^32^, ^33^, ^34^ Rats were placed onto a platform (8 cm in diameter) inside a 75.5 cm × 50.5 cm × 45 cm water tank, which was filled with water up to 1 cm below the level of the platform. In this paradigm, rats could not completely relax the large muscle groups (a necessary condition for REM sleep to occur) without falling off the platform, becoming wet and awake. Sleep deprivation was conducted from ZT1.5 (8:30) to ZT7.5 (14:30) each day for 7 consecutive days.

### 2.3 Behavioral studies

#### 2.3.1 Novel object location

The arena was 110 cm × 110 cm with 50 cm high walls; black shapes on all four walls provided spatial and contextual cues. Animals were initially habituated to an arena for 10 min each day for consecutive 3 days. Object exploration and novel location testing occurred 30 min after the last habituation. Essentially, rats explored two identical objects in the arena for 5 min (object exploration). The exploration time of familiar object A1 and familiar object A2 was compared to evaluate whether there was a preference for the location of the object. Rats were returned to their home cages for a 30‐min rest period. Finally, during the 5‐min test, rats explored the arena with the same two objects: one in the same location (familiar) and the other moved to a different location in the arena (novel). The exploration times of rats for novel object location (*N*) and original familiar object location (*F*) during the test period were recorded. The calculated recognition index [(RI) = *N*/(*N* + *F*) × 100%] was positively correlated with the spatial memory of rats. The arena was cleaned with 75% ethanol between trials to eliminate olfactory cues.

#### 2.3.2 Novel object recognition

The apparatus for the novel object recognition task was the same as used for the novel object location task. Two objects were always located in the back corners of the box. The location and objects were counterbalanced to control for any preferences that the rats might have had for one of the corners or of the objects. This behavioral procedure involved two phases: training and test trials. During training and test sessions, animals were placed at the center of the arena and exploratory behavior toward both objects was recorded for 5 min. The arena was cleaned with 75% ethanol between trials to eliminate olfactory cues. The training session was performed in the presence of two identical objects. For the test session, carried out 30 min after training, one of the two objects used in the training session was replaced by a novel object. The exploration times of rats for novel (*N*) and original familiar object (*F*) during the test period were recorded and the RI was calculated [RI = *N*/(*N* + *F*) × 100%].

#### 2.3.3 Morris water maze

The Morris water maze (MWM) was used to evaluate spatial learning using a modified Whishaw protocol.^35^ The water maze was a black round water pool (diameter:1.2 m, height: 0.6 m) in which a platform (diameter: 8 cm, height: 23 cm) was located 2 cm below the water surface in the center of one of the four quadrants. The pool was filled with water (22–26°C, depth: 25 cm). The water pool and the surrounding environment were kept the same during the whole experiment. At the beginning of the experiment, all of the rats were trained in the MWM. For each training, the rat was placed into water at one of four quadrants around the pool's perimeter, facing the wall. Once the rat found the platform (escape latency) in 60 s, the training was terminated. Otherwise, the rat was guided to the platform and allowed to stay for 10 s. If a rat found and stood on the platform within 60 s for continuous four times, it was considered to pass the water maze training. Rats who failed after 16 times training (four times at each entrance point) were excluded from our study. In the water maze test, if the rat did not find the platform within 60 s, a value of 60 s was assigned as escape latency. The average datum of escape latency (s) over four times was taken as the result. After the last test, each rat was subjected to a probe trial (60 s) to assess spatial memory in which the platform was removed. The number of crossings over the former platform location, the swimming time ratio, and the swimming distance ratio of the real platform quadrant within 60 s were recorded.

### 2.4 RNA isolation and quantitative real‐time PCR

Total RNA from plasma and the hippocampus were extracted by TRIZOL® reagent (Invitrogen) according to the manufactory manual. The RNA samples were dissolved in 50 μL of DEPC‐H_2_O and subsequently stored at −80°C. The quantification and purity of the RNA were measured by NanoDrop2000 (Thermo‐Fisher Scientific). Total RNA was transcribed by a HiFiScript cDNA Synthesis Kit (CWBIO) according to the manufactory protocol. Real‐time PCR was performed on the ABI7300 platform (Applied Biosystems, Life Technologies) by using ChamQ SYBR qPCR Master Mix (Vazyme). Data were analyzed using the 2‐ΔΔCt method. Primers for each RNA were listed in Table S1.

### 2.5 Protein extraction and Western blotting

100 mg of hippocampus was homogenized in 1 mL of protein lysate buffer (CWBIO) with 1 × protease inhibitor (EDTA free, Roche), sonicated 1 min on ice, and then cleared by centrifugation at 12,000*g* for 20 min at 4°C. The supernatant was stored at −80°C. Protein concentrations were analyzed using a NanoDrop2000 (Thermo‐Fisher Scientific). 20 μg protein was loaded and run through a 10% sodium dodecyl sulfate‐polyacrylamide gel for electrophoresis and transferred to a nitrocellulose membrane to perform the Western blot analysis. Primary antibodies used were Anti‐Bmal1 (1:1000, Bioss), Anti‐Clock (1:1000, Affinity Biosciences), Anti‐HDAC3 (1:1000, Bioss), anti‐GAPDH (1:10,000, Bioss). Appropriate protein bands were detected by enhanced chemiluminescence (CWBIO) and visualized by Image J software (Rawak Software Inc.). Each band signal was quantified with Image J software and normalized to internal control. All processes were triplicated.

### 2.6 Statistical analysis

All analyses were performed with GraphPad Prism 8 (GraphPad Software). Data were presented as the mean ± standard error (SEM). The Shapiro–Wilk test was used to test the normality. Repeated‐measures analysis of variance (ANOVA) followed by Tukey's post hoc multiple comparison tests to determine differences in escape latency and distance traveling of MWM and mRNA levels between study groups (with group and time as factors). One‐way ANOVA followed by Bonferroni post hoc test was applied to analyze differences in other behavior tests and protein levels among groups. Pearson correlation analysis was applied to evaluate the relationship between protein levels. *p* < 0.05 were considered statistically significant.

## 3 RESULTS

### 3.1 Chronic REM sleep deprivation selectively impaired hippocampal‐dependent spatial learning and memory

**FIGURE 2:**
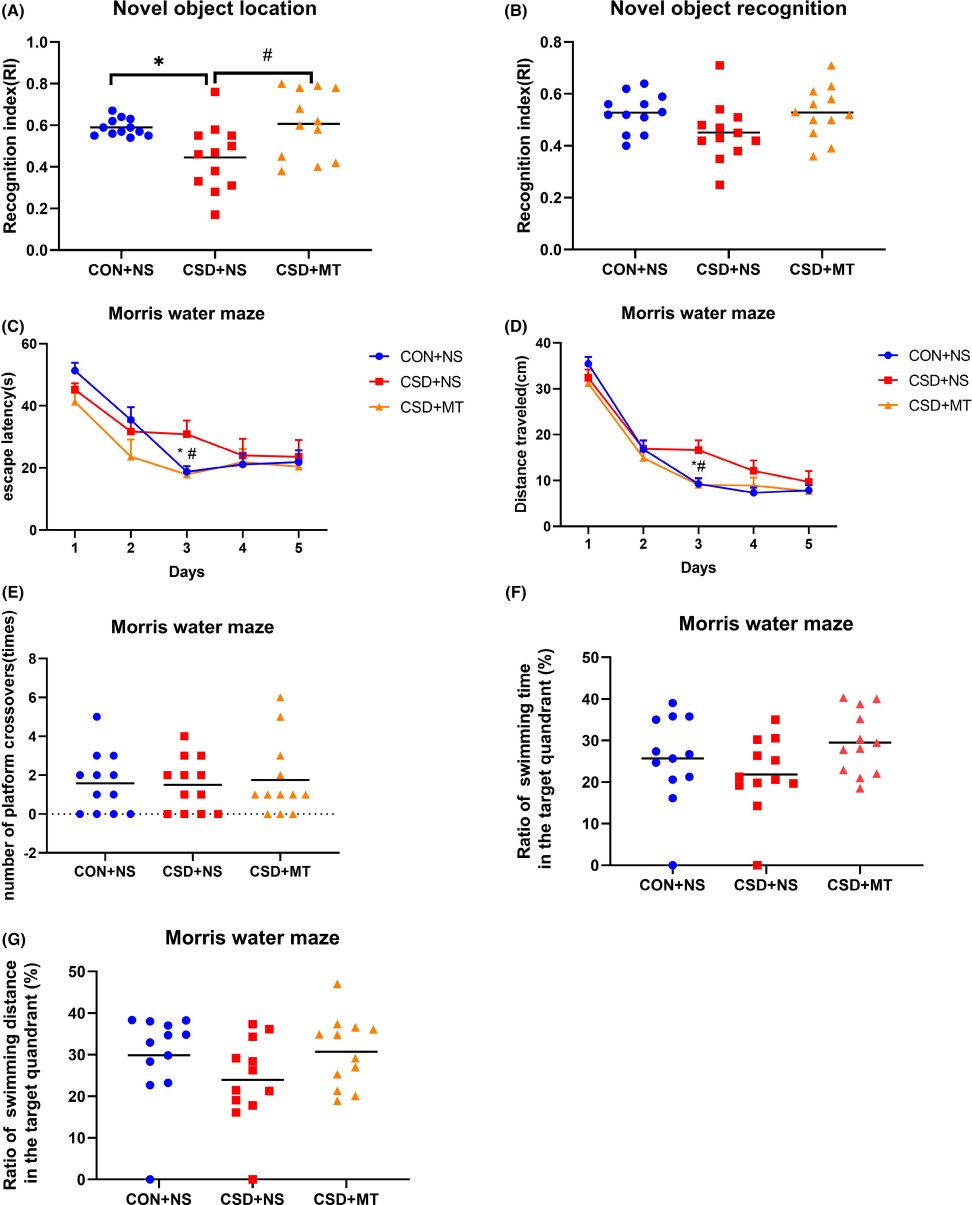
Melatonin attenuated chronic REM sleep deprivation‐induced hippocampal‐dependent spatial learning and memory deficits. Chronic REM sleep deprivation (CRSD) significantly decreased recognition index (RI) in novel object location (A) but did not affect novel object recognition (B) compared to control (CON + NS). In the Morris water maze (MWM), CRSD extended escape latency (C) and distance traveling (D) on the third day compared to control while the numbers of platform crossing (E), the swimming time ratio (F), and the swimming distance ratio (G) of the real platform quadrant in MWM were not significantly different between CRSD + NS group and CON + NS group. Melatonin (MT) treatment specifically attenuated CRSD‐induced deficits on novel object location (A) and (C, D) MWM, but had no effects on novel object recognition (B). *N* = 12 for each group. *p < 0.05 CRSD + NS compared to CON + NS; #*p* < 0.05 CRSD + MT compared to CRSD + NS.

In this study, a significantly lower RI in novel object location (Figure 2A, *p* = 0.032) was observed in the CRSD + NS group compared to the CON + NS group. In MWM, longer escape latency (Figure 2C, *p* = 0.046) and distance traveling (Figure 2D, *p* = 0.023) were observed in the CRSD + NS group only on the third day. No statistical significance was found on the numbers of platform crossing (Figure 2E, *p* = 0.99), the swimming time ratio (Figure 2F, *p* = 0.53), and the swimming distance ratio (Figure 2G, *p* = 0.29) of the real platform quadrant in MWM between the CRSD + NS group and the CON + NS group. There was no significant difference in the RI in novel object recognition between the CRSD + NS group and the CON + NS group (Figure 2B, *p* = 0.15). These data suggested that CRSD prefers to impair hippocampal‐dependent spatial learning and memory, which were consistent with previous studies.^36^, ^37^

### 3.2 Melatonin attenuated chronic REM sleep deprivation‐induced cognitive deficient

With melatonin administration, the decrease in RI of novel object location by CRSD was significantly attenuated (Figure 2A, *p* = 0.017). The up‐regulated escape latency and distance traveling of MWM on the third day by CRSD were also modified by melatonin treatment (for escape latency, *p* = 0.039, Figure 2C; for distance traveling *p* = 0.012, Figure 2D). There was no significant difference on novel object recognition tests between CRSD + MT group and CRSD + NS group (Figure 2B, *p* = 0.16). Also, there was no statistical difference on numbers of platform crossing (Figure 2E, *p* = 0.91), the swimming time ratio (Figure 2F, *p* = 0.095), and the swimming distance ratio (Figure 2G, *p* = 0.19) of the real platform quadrant in MWM between CRSD + MT group and CRSD + NS group. These data indicated melatonin attenuated CRSD‐induced hippocampal‐dependent spatial learning and memory impairment.

### 3.3 Melatonin prevented chronic REM sleep deprivation‐induced circadian arrhythmia in Hippocampus

Previous studies had shown a 6‐h SD resulting in a strong reduction of neuronal activity in the SCN.^38^ Hence, we determined whether CRSD affects circadian rhythms in both hippocampus and peripheral blood. In addition, HDAC3, which has been reported to maintain a robust circadian clock and involve cognition regulation,^39^ was also assessed in this study. The dynamic levels of core *clock genes* (*Bmal1*, *Clock*, *Cry1*, *Cry2*, *Per1*, and *Per2*) as well as *HDAC3* in 24 h were measured in the CRSD + NS, CON + NS as well as CRSD + MT group by quantitative real‐time PCR.

**FIGURE 3:**
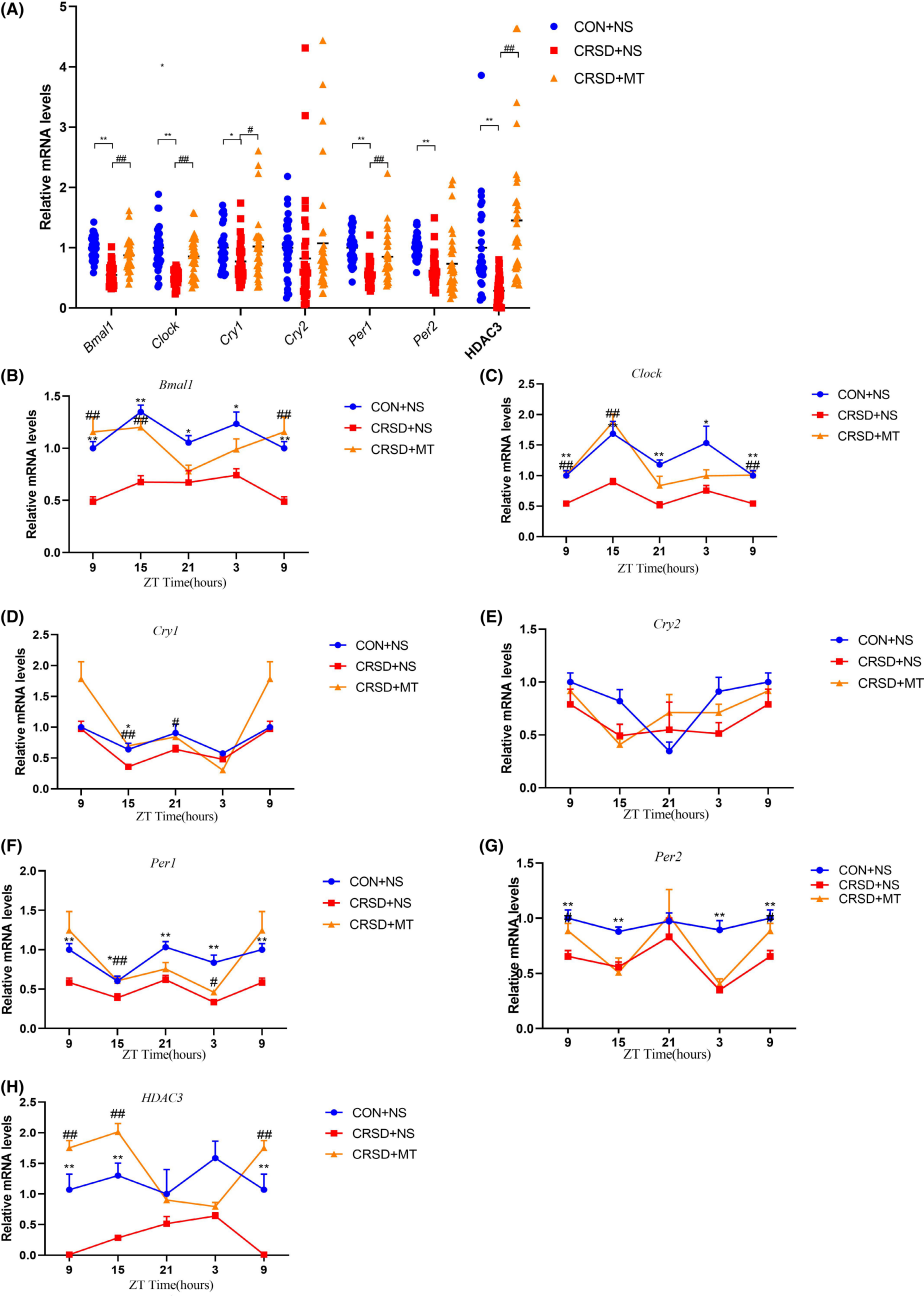
Effects of chronic REM sleep deprivation and melatonin on *clock* genes and *HDAC3* gene expression in the hippocampus. (A) The general levels of *clock* genes and *HDAC3* in the hippocampus among groups. Chronic REM sleep deprivation (CRSD) reduced the general levels of *Bmal1*, *Clock*, *Per1*, *Per2*, and *HDAC3* compared to control (CON + NS) and these decreases were prevented by melatonin (MT) treatment. The *Cry1* expression was not affected by CRSD but was up‐regulated by melatonin treatment. The levels of *Cry2* were not significantly changed by CRSD or melatonin. (B–H) The dynamic changes of *clock* genes and *HDAC3* at different time points in the hippocampus among groups. All these detected *clock genes* (B–G) had intrinsic rhythms, but the *HDAC3* (H) had little fluctuation at different time points in the control group. The amplitude of *Bmal1* (B) and *Clock* (C) was globally down‐regulated by CRSD with stable inherent oscillation compared to control and melatonin treatment reversed these decreases. The amplitude of *Per1* (F), *Per2* (G), and *Cry2* (E) was altered by CRSD at certain time points with the disappearance of inherent oscillation compared to the control. Melatonin prevented CRSD‐induced down‐regulation of *Per1* (F) and *Per2* (G) but had no significant effect on *Cry2* (E). CRSD has little effect on the amplitude or phases of *Cry1* but melatonin treatment up‐regulated *Cry1* levels at certain peak times with inherent oscillation (D). CRSD significantly reduced *HDCA3* amplitude mostly at ZT15 and melatonin up‐regulated *HDAC3* at several time points (H). Data are presented as mean ± SE. *N* = 12 for each group,*p < 0.05, **p < 0.01 CRSD + NS compared to CON + NS; #*p* < 0.05, ##*p* < 0.01 CRSD + MT compared to CRSD + NS.

The general levels of *Bmal1*, *Clock*, *Per1*, *Per2*, and *HDAC3* were reduced by CRSD compared to control and these decreases were prevented by melatonin treatment in the hippocampus (Figure 3A, all *p* < 0.05). The *Cry1* expression was not affected by CRSD but up‐regulated by melatonin treatment (Figure 3A). The levels of *Cry2* were not significantly changed by CRSD nor melatonin treatment (Figure 3A, *p* < 0.05). On the dynamic process in the hippocampus, all these detected *clock genes* had intrinsic rhythms in the control group (Figure 3B–G, all *p* < 0.05). Following CRSD, the amplitude of *Bmal1* and *Clock* was globally down‐regulated in all time points compared to control with relatively stable phases of peak and valley (Figure 3B,C, *p* < 0.05). While the amplitude of *Per1*, *Per2*, and *Cry2* in CRSD + NS was altered at certain time points with the disappearance of inherent oscillation compared to the control (Figure 3E–G). There was no statistical change in the amplitude or phases of *Cry1* in the CRSD + NS group compared to the CON + NS (Figure 3D, all *p* > 0.05). Different from *core clocks*, the basic levels of *HDAC3* had little fluctuation at different time points in the CON + NS group (Figure 3H, *p* > 0.05). CRSD significantly reduced hippocampal *HDCA3* mostly at ZT15 compared to the CON + NS group (Figure 3H, *p* < 0.001). With the melatonin administration, the decreased amplitude of *Bmal1*, *Clock*, *Per1*, *Per2*, and *HDAC3* by CRSD was attenuated at different time points (Figure 3B,C,F,G, all *p* < 0.05). Moreover, the disrupted time phases of *Per1* by CRSD were also recovered. Interestingly, although CRSD has little effect on the amplitude and phases of *Cry1*, melatonin treatment up‐regulated the *Cry1* levels at a certain peak time with inherent oscillation (Figure 3D, *p* < 0.05). There was no significant effect on *Cry2* by melatonin treatment (Figure 3E, *p* > 0.05).

### 3.4 Melatonin prevented chronic REM sleep deprivation‐induced circadian arrhythmia in peripheral blood

**FIGURE 4:**
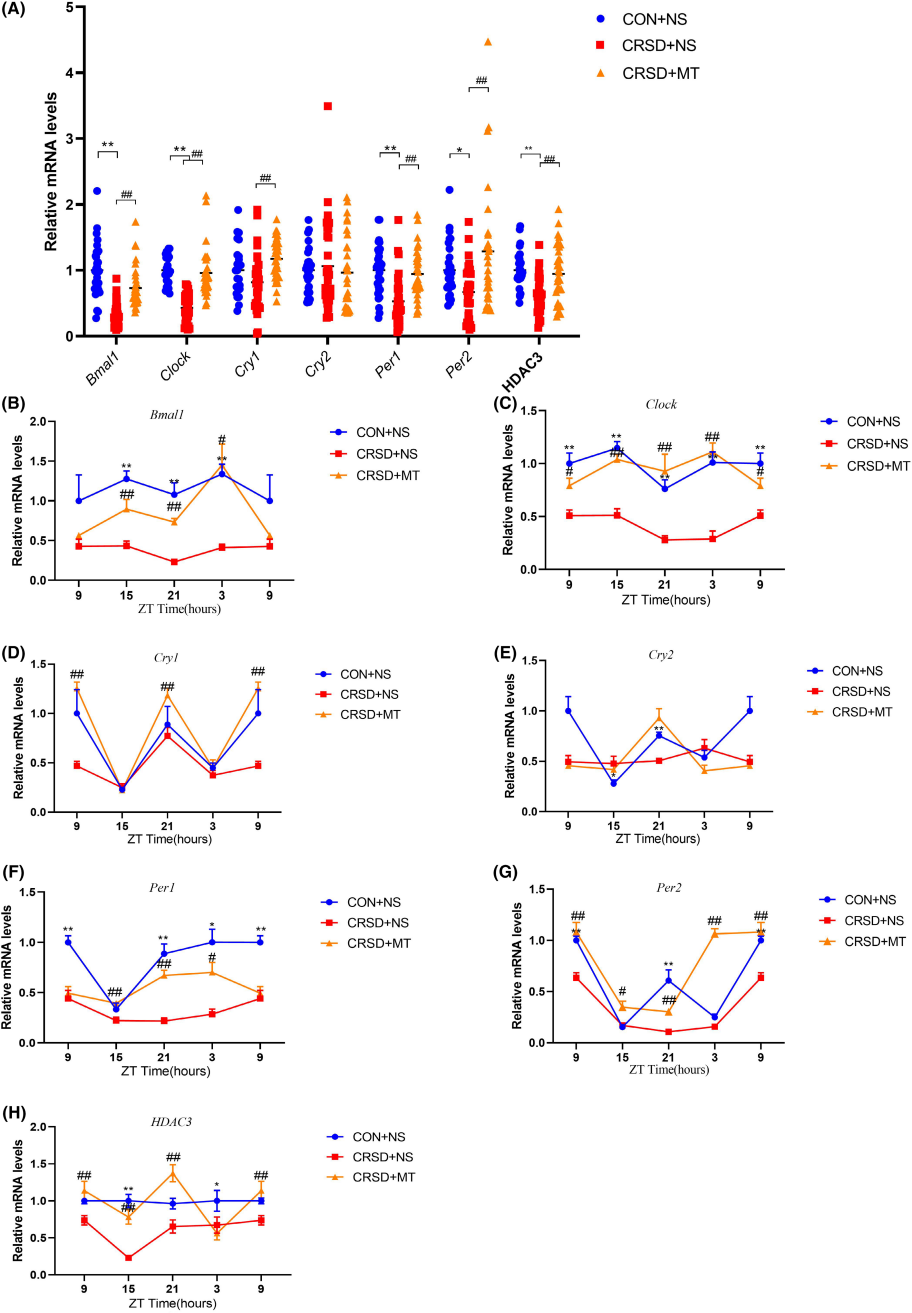
Effects of chronic REM sleep deprivation and melatonin on *clock* genes and *HDAC3* gene expression in the peripheral blood. (A) The general levels of *clock* genes and *HDAC3* in peripheral blood among groups. Chronic REM sleep deprivation (CRSD) reduced the general levels of *Bmal1*, *Clock*, *Cry1*, *Per1*, *Per2*, and *HDAC3* compared to control (CON + NS) and these decreases were prevented by melatonin (MT) treatment except for *Per2*. There was no significant change in *Cry2* among groups. (B–H) The dynamic changes of *clock* genes and *HDAC3* at different time points in peripheral blood among groups. The amplitude of *Bmal1* (B), *Clock* (C), *Per1* (F), *Per2* (G), and *HDAC3* (H) was globally down‐regulated by CRSD with stable inherent oscillation compared to control (CON + NS). Melatonin (MT) treatment prevented CRSD‐induced decreases in *Bmal1* (B), *Clock* (C), *Per1* (F), and *HDAC3* (H) amplitude at certain time points while the down‐regulations of *Per2* (G) were not affected. Although CRSD had a limited effect on *Cry1*, melatonin treatment up‐regulated the *Cry1* levels at certain time points (D). Neither CRSD nor melatonin had a significant effect on *Cry2* (E). Data are presented as mean ± SE. *N* = 12 for each group, *p < 0.05, **p < 0.01 CRSD + NS compared to CON + NS; #*p* < 0.05, ##*p* < 0.01 CRSD+MT compared to CRSD + NS.

Similar to the hippocampus, the general levels of *Bmal1*, *Clock*, *Cry1*, *Per1*, *Per2*, and *HDAC3 in peripheral blood* were reduced by CRSD compared to control and these decreases were prevented by melatonin treatment except for *Per2* (Figure 4A, all *p* < 0.05). The levels of *Cry2* were not significantly changed by CRSD or melatonin treatment (Figure 4A, *p* > 0.05). On the dynamic process in peripheral blood, all these detected *clock genes* as well as *HDAC3* mRNA had inherent oscillation in the control group (Figure 4B–H). The amplitude of *Bmal1*, *Clock*, *Per1*, *Per2*, and *HDAC3* was globally down‐regulated in the CRSD + NS group with stable inherent oscillation (Figure 4B,C,F,G,H, all *p* < 0.05). There was a limited effect of CRSD on the amplitude or phases of *Cry1* and *Cry2* compared to the control (Figure 4D,E, all *p* > 0.05). Melatonin treatment prevented the CRSD‐induced decreases in the amplitude of *Bmal1*, *Clock*, *Per1*, and *HDAC3* at certain time points (Figure 4B,C,F,H) while had no significant effect on *Per2* (Figure 4G). Although CRSD had little effect on *Cry1*, melatonin treatment up‐regulated the *Cry1* levels at certain time points in peripheral blood (Figure 4D). No significant effect of melatonin treatment was observed on *Cry2* (Figure 4E, *p* > 0.05). These data indicate *HDAC3* and core clocks of *Bmal1* and *Clock* mRNA are affected by CRSD, and melatonin treatment modified these CRSD‐induced alterations in both central and peripheral blood.

### 3.5 Melatonin reversed alterations of hippocampal clocks and HDAC3 protein levels from chronic REM sleep deprivation

**FIGURE 5:**
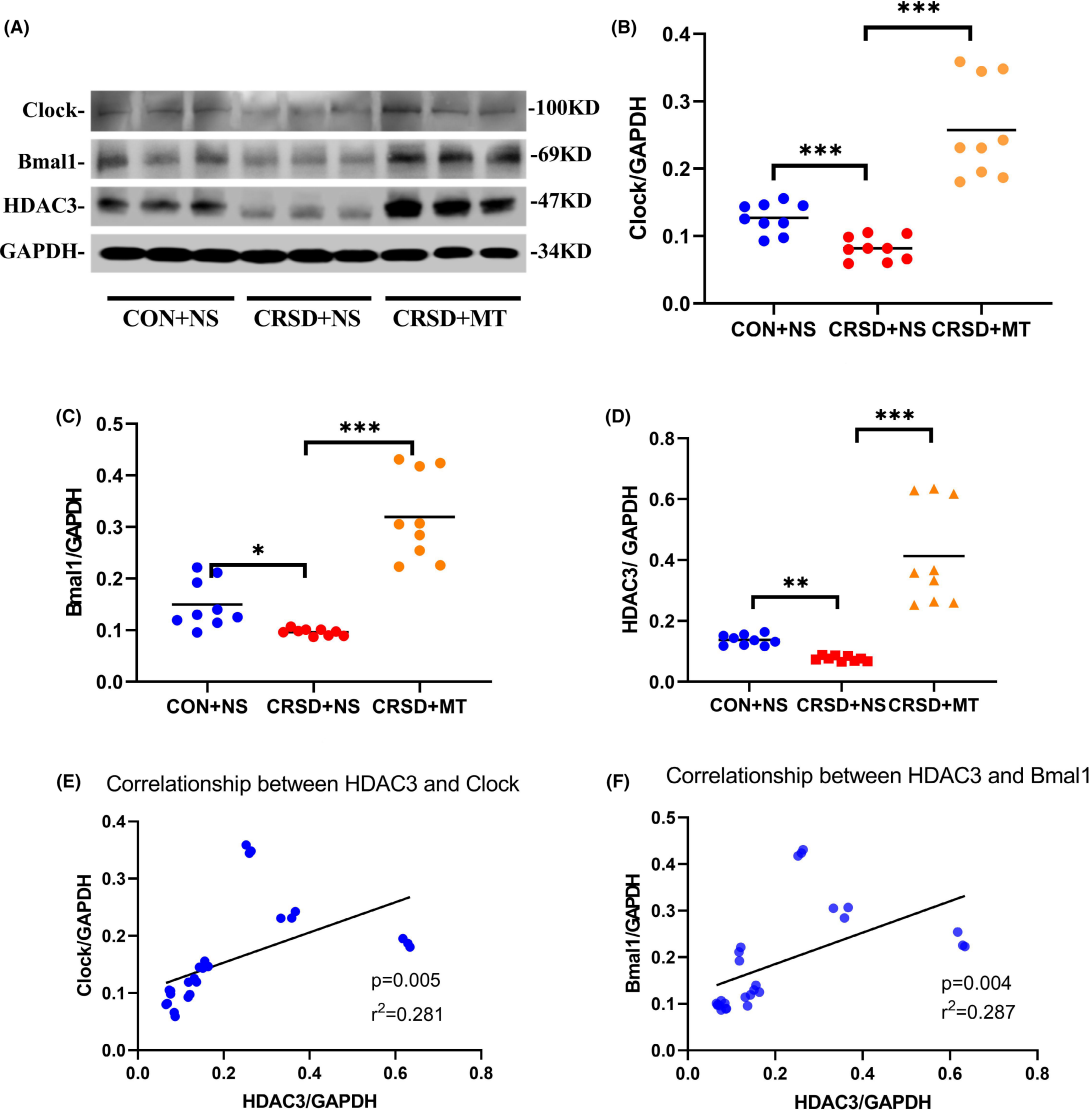
Effects of chronic REM sleep deprivation and melatonin on Bmal1 and HDAC3 protein in the hippocampus. (A) Representative western blotting blots of Clock, Bmal1, and HDAC3 in the hippocampus of control (CON + NS), sleep deprivation (CRSD + NS), and melatonin treatment (CRSD + MT) groups. (B) Quantitative analysis of Bmal1 protein levels among three groups. (C) Quantitative analysis of Bmal1 protein levels among three groups. (D) Quantitative analysis of HDAC3 protein levels among three groups. (D) Pearson correlation analysis on Clock and HDAC3 protein levels in the whole cohort. (E) Pearson correlation analysis on Bmal1 and HDAC3 protein levels in the whole cohort. Data are presented as mean ± SE from three independent experiments. *N* = 9 for each group, *p < 0.05, **p < 0.01, ***p < 0.001.

As the *Clock*, *Bmal1* and *HDAC3* mRNA were significantly changed by CRSD and melatonin treatment, their protein levels in the hippocampus were further evaluated in the CRSD + NS group, CON + NS group as well as CRSD + MT group. The expression of Clock, Bmal1, and HDAC3 was all significantly decreased by CRSD compared to the CON + NS (Figure 5A–D, for Clock *p* < 0.001; for Bmal1 *p* = 0.024; for HDAC3 *p* = 0.007). With pretreatment of melatonin, CRSD‐induced alterations of these three proteins were reversed (Figure 5A–D, for Clock *p* < 0.001; for Bmal1 *p* < 0.001; for HDAC3, *p* < 0.001). Pearson correlation analysis revealed significant correlations between hippocampal HDAC3 levels and Bmal1 as well as Clock levels in all cohorts (Figure 5E,F, for HDAC3 and Clock *p* = 0.005, coefficients = 0.53; for HDAC3 and Bmal1 *p* = 0.004, coefficients = 0.54).

## 4 DISCUSSION

**FIGURE 6:**
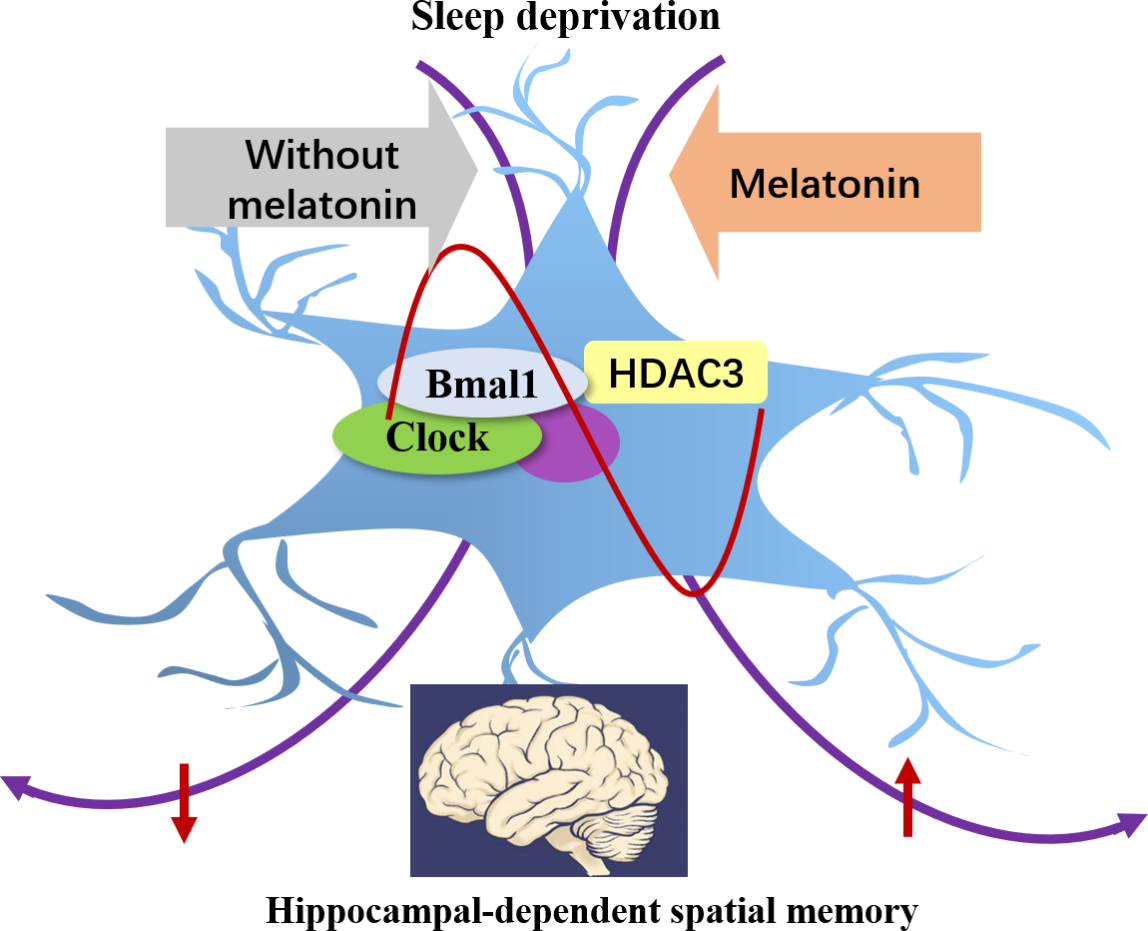
Proposed model for melatonin to attenuate chronic REM sleep deprivation‐induced cognitive deficits via regulating HDAC3–Bmal1 pathway.

It has been widely accepted that sleep plays a critical role in regulating cognition, but the underlying mechanism remains poorly understood and there is a lack of effective treatment for dementia. In the current study, we identified that pretreatment with melatonin significantly attenuated CRSD‐induced intermediate‐term and long‐term spatial learning and memory impairment, and this protective effect may attribute to the regulation of HDAC3 and Bmal1/Clock complex (Figure 6).

REM sleep and NREM sleep are vital for supporting normal cognition but may play roles through different ways. Although considerable studies have shown that REM sleep deprivation results in emotional and spatial memory impairment,^40^, ^41^ some studies report that acute REM sleep deprivation enhances avoidance learning and spatial memory^42^ and reinstates memory retrieval in mice.^43^ In this study, we observed significant impairment in novel object location and hidden platform MWM following CRSD, but no significant changes were found in novel object recognition. Among cognitive behavior tests, novel object location and novel object recognition are tests of intermediate‐term memory while MWM is a test of long‐term memory.^44^ Novel object location assay and hidden platform MWM are almost exclusively dependent on the hippocampus while novel object recognition is also involved additional brain regions such as the amygdala and perirhinal cortex.^44^ Hence, our data indicate CRSD selectively injures intermediate‐term and long‐term hippocampal‐dependent spatial learning and working memory.

Sleep deprivation was historically assumed to have major effects on the homeostatic process but minor or no effects on the circadian pacemaker. The circadian rhythms are controlled by a self‐sustained network of cellular clocks built from interlocked transcriptional–translational feedback loops. At its most fundamental level, this loop is centered on a basic helix–loop–helix transcription factor formed by *Clock* and *Bmal1*, starting in subjective day.^45^ The complex serves as a transcriptional activator of *Per* and *Cry*. The Per and Cry proteins accumulate in the cytosol, reaching a critical concentration in the late afternoon and repress the transactivation of the Bmal1/Clock complex in the nucleus, forming a negative feedback loop.^45^ In this study, we found the mRNAs of *Clock* and *Bmal1* were globally down‐regulated in amplitude by CRSD with constant intrinsic oscillation in both hippocampus and peripheral blood. Moreover, the protein levels of Bmal1 and Clock were also significantly reduced by CRSD. Our findings indicate that sleep deprivation not only influences sleep homeostatic responses but also affects circadian regulation, which is in agreement with some recent studies.^38^, ^46^, ^47^ The phasing, periodicity, and amplitude of molecular rhythms can be influenced by a wide neural plasticity array of intracellular effectors. Bmal1/Clock complex‐mediated activation of CCGs was coupled to circadian changes in histone acetylation at their promoters^39^ and the finding of a circadian HAT opened the search for a counterbalancing HDACs. HDAC3, the most highly expressed class I HDAC in the brain, has been shown as a critical component of the core mammalian circadian negative feedback loop by regulating both the activation and repression processes.^48^ Genetic depletion of *HDAC3* results in low amplitude of circadian rhythms and Bmal1 protein levels.^48^ In this study, we observed CRSD‐induced remarkable decreases in hippocampal HDAC3 levels accompanied by down‐regulation of Bmal1/Clock expression. Additionally, the levels of Bmal1/Clock and HDAC3 were significantly correlated. The distinct role of HDAC3 in cognition was under debate from previous studies. Although deletion or selective inhibition of HDAC3 has been proposed as a promising intervention to improve memory and neural plasticity,^28^, ^49^, ^50^ the presence of severe cytotoxicity and cognitive impairment in *Hdac3*‐deleted mice challenged its therapeutic effect.^26^, ^51^ As HDAC3 has been reported to regulate both the activation and repression processes of the circadian negative feedback loop,^48^ we propose those conflicting results could be partially explained by the opposing roles of HDAC3 on the circadian cycle.

Interestingly, pretreatment with melatonin was found to ameliorate CRSD‐induced cognitive impairment as well as HDAC3 and Bmal1/Clock interruption in this study.

As the phase marker of the circadian pacemaker, melatonin is believed to closely associate with sleep and has been clinically recommended for treating sleep disorders. But its effect on cognition remains poorly understood. Deficient in Bmal1 has been shown to cause learning and memory impairment^7^ and melatonin promotes cell survival by up‐regulating the expression of Bmal1.^52^ In addition, melatonin increases cell proliferation via inducing HDAC3 and clock genes in high fat diet mice model.^9^ Hence, we propose that CRSD reduces hippocampal HDAC3 levels in rats, which further down‐regulates the expression of the Bmal1/Clock complex and results in the impairment of hippocampal‐dependent spatial learning and working memory. Melatonin treatment attenuates CRSD‐induced cognitive impairment via regulating HDAC3‐Bmal1/Clock interaction.

Altogether, our data indicate decreases in HDAC3 and Bmal1/Clock complex could play critical roles in the pathogenesis of CRSD‐induced hippocampal‐dependent spatial memory impairment. Chronic melatonin pretreatment reverses the interruption of HDAC3 and circadian rhythms from CRSD, which eventually ameliorates cognitive deficits (Figure 6). These findings not only explore a broader understanding of sleep and cognition but also provide a potential new therapeutic target for sleep interruption‐related cognitive impairment.

## AUTHOR CONTRIBUTIONS

Jinxia Zhou and Guoshuai Yang contributed to the conception and design of the study. Yujie Hu performed the experiments. Yefan Lv organized the database. Yujie Hu and Xiaoyan Long performed the statistical analysis. Yujie Hu wrote the first draft of the manuscript. Jinxia Zhou and Guoshuai Yang supervised all studies and drafting of the manuscript. All authors who contributed to the manuscript have read and approved the submitted version.

## CONFLICT OF INTEREST STATEMENT

The authors declare no conflict of interest.

## Supporting information


Data S1.

Table S1.

## Data Availability

The data that support the findings of this study are available from the corresponding author upon reasonable request.
