# Supplementary material for: Melatonin attenuates chronic sleep deprivation‐induced cognitive deficits and HDAC3‐Bmal1/clock interruption
Source: CNS Neurosci Ther. 2023 Sep 18;30(3):e14474. doi: 10.1111/cns.14474 (PMC10916425; doi:10.1111/cns.14474)
Supplement: Supplementary file 1 — Data S1. [file CNS-30-e14474-s002.pdf]

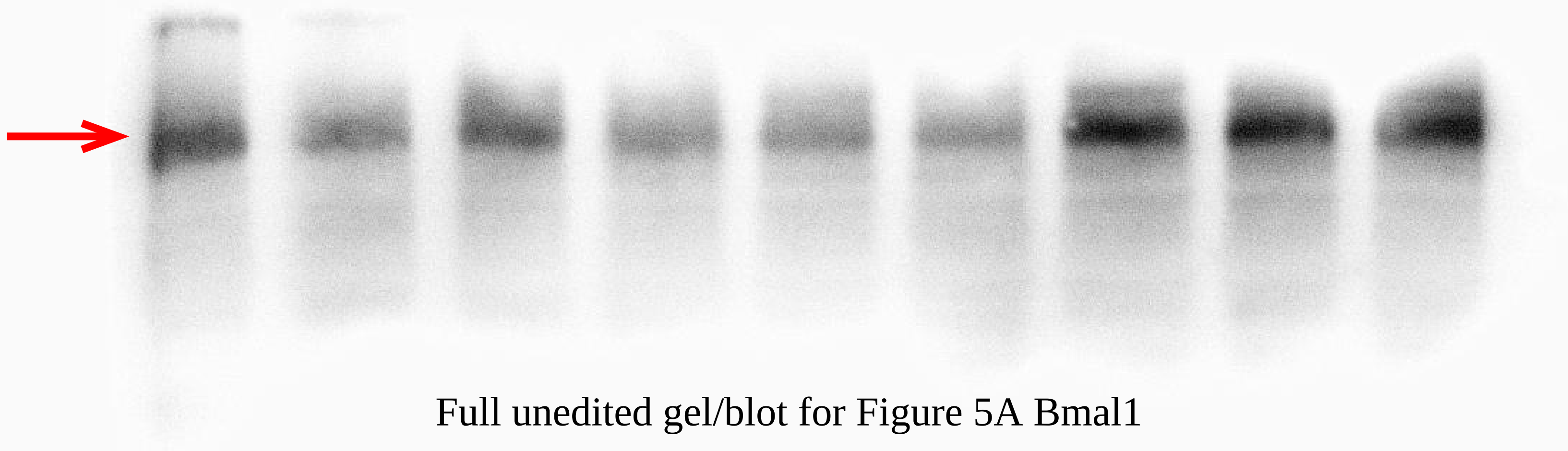

Full unedited gel/blot for Figure 5A Bmal1

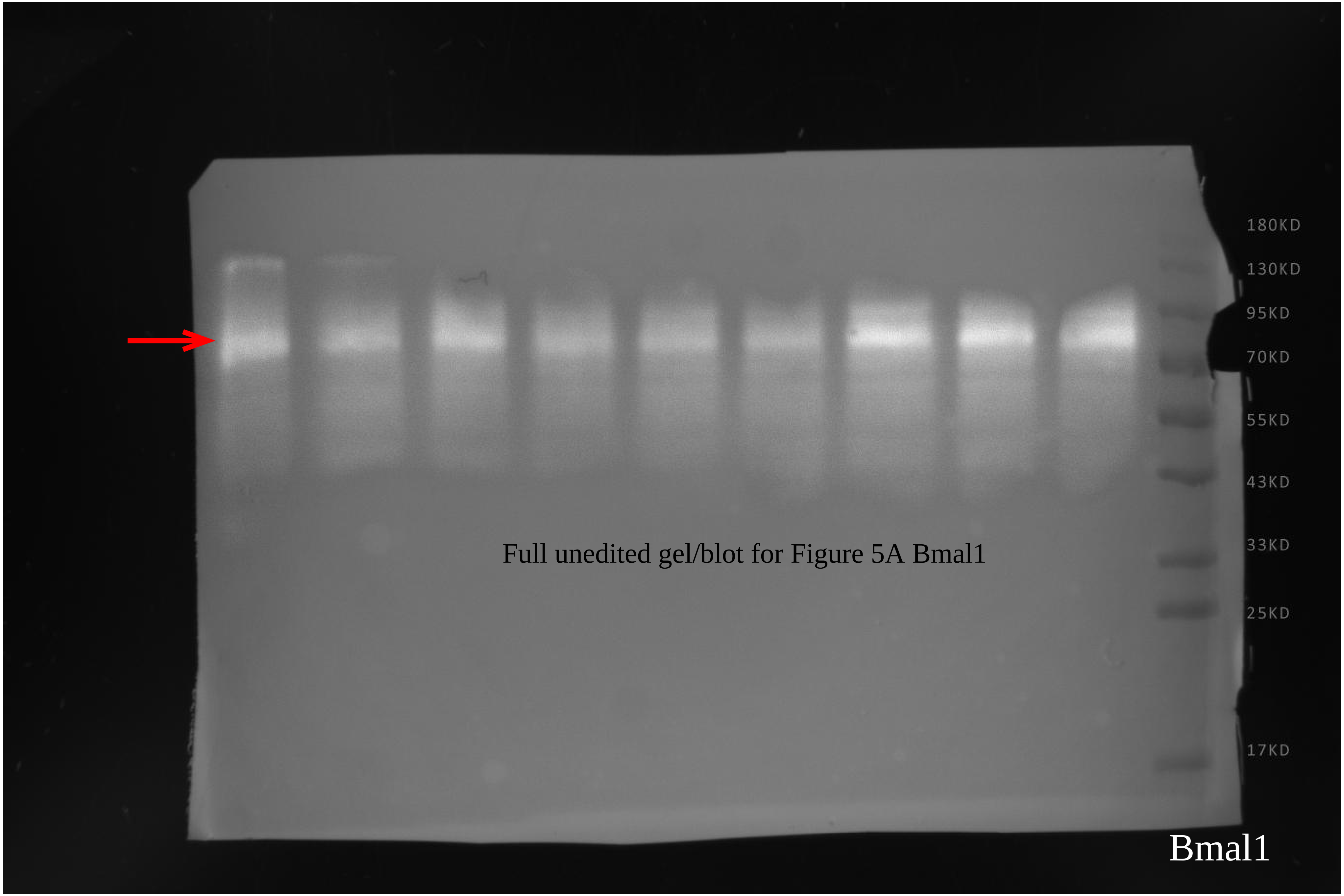

Full unedited gel/blot for Figure 5A Bmal1

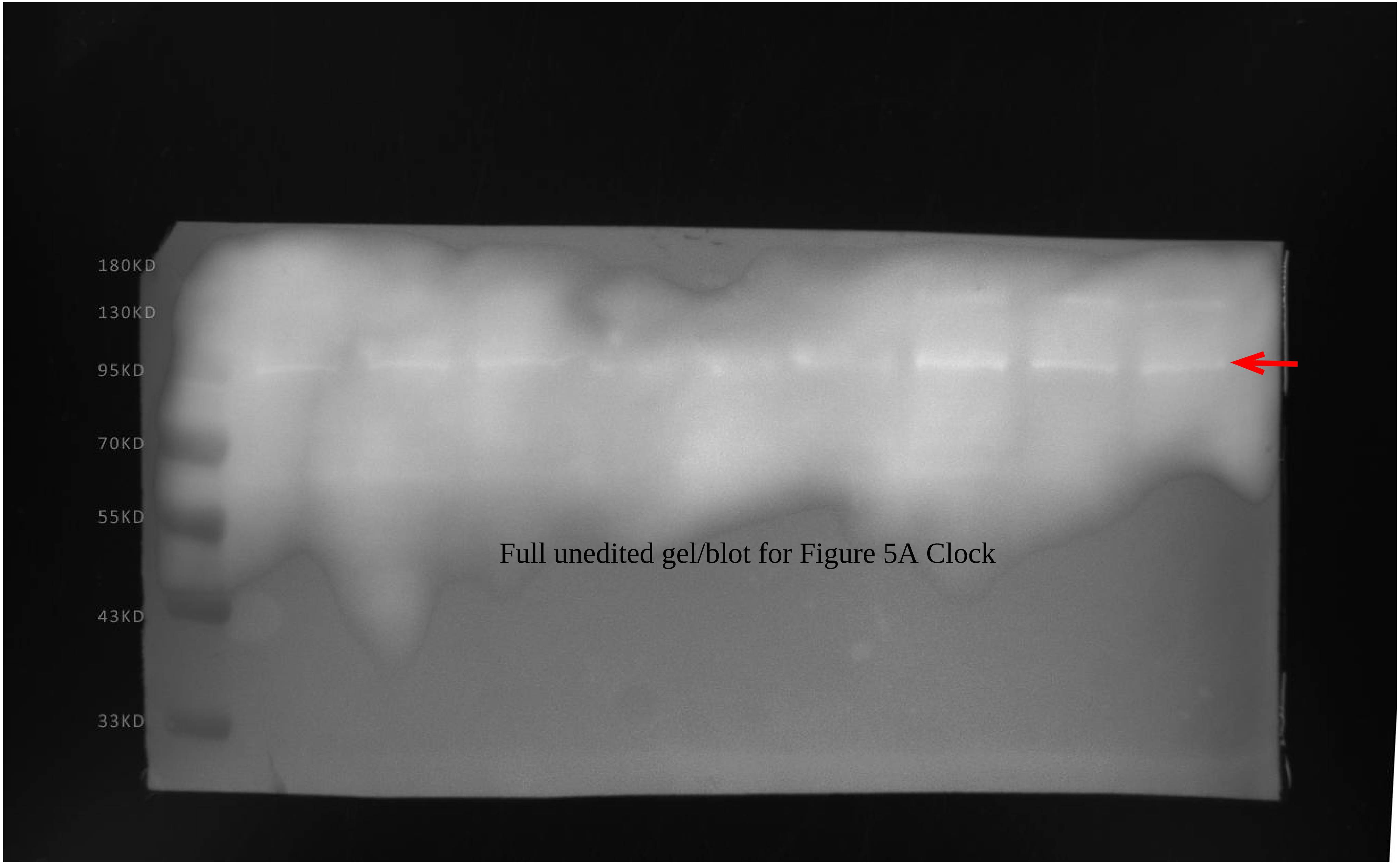

Full unedited gel/blot for Figure 5A Clock

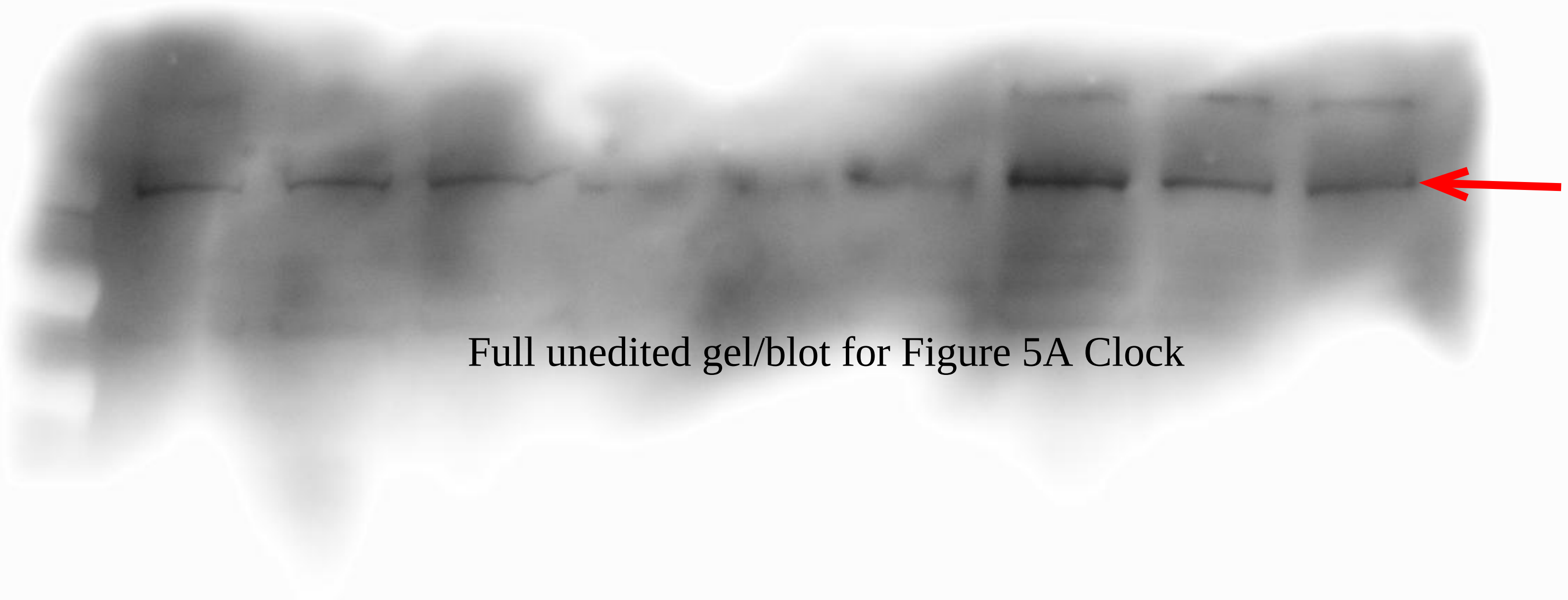

Full unedited gel/blot for Figure 5A Clock

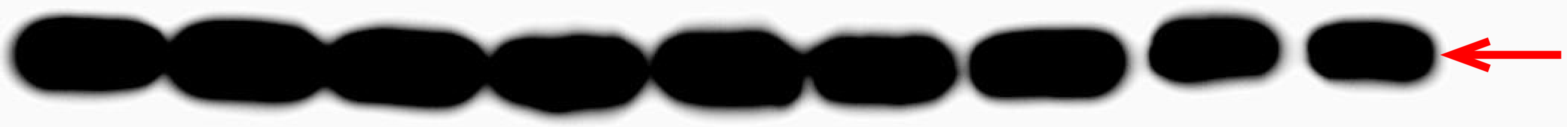

Full unedited gel/blot for Figure 5A GAPDH

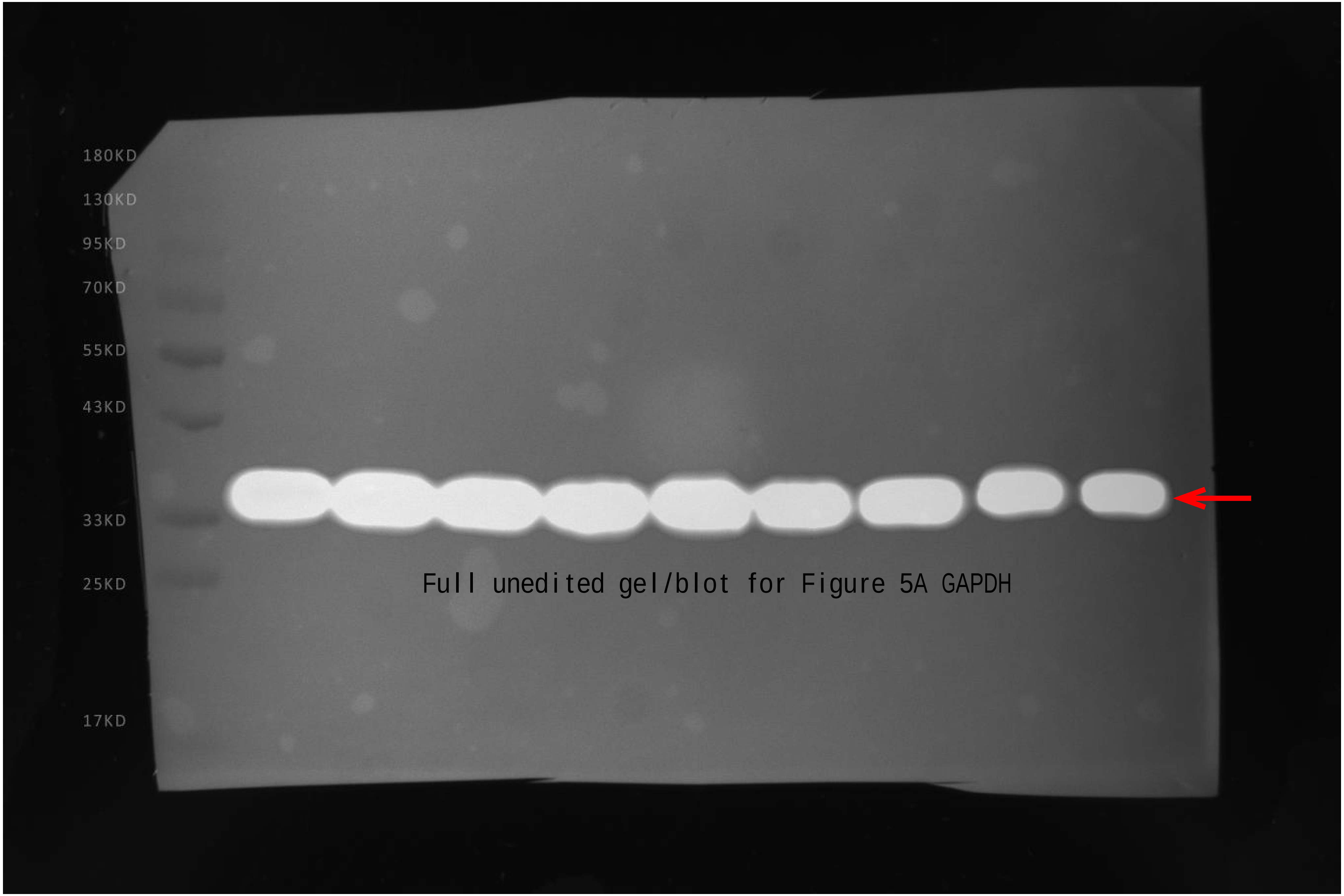
180KD
130KD
95KD
70KD
55KD
43KD
33KD
25KD
17KD
Full unedited gel/blot for Figure 5A GAPDH

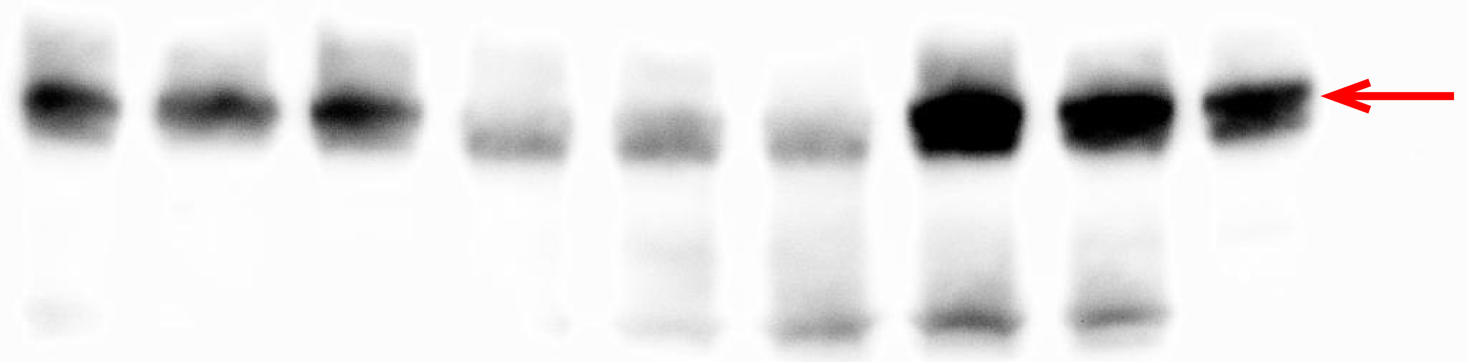

Full unedited gel/blot for Figure 5A HDAC3

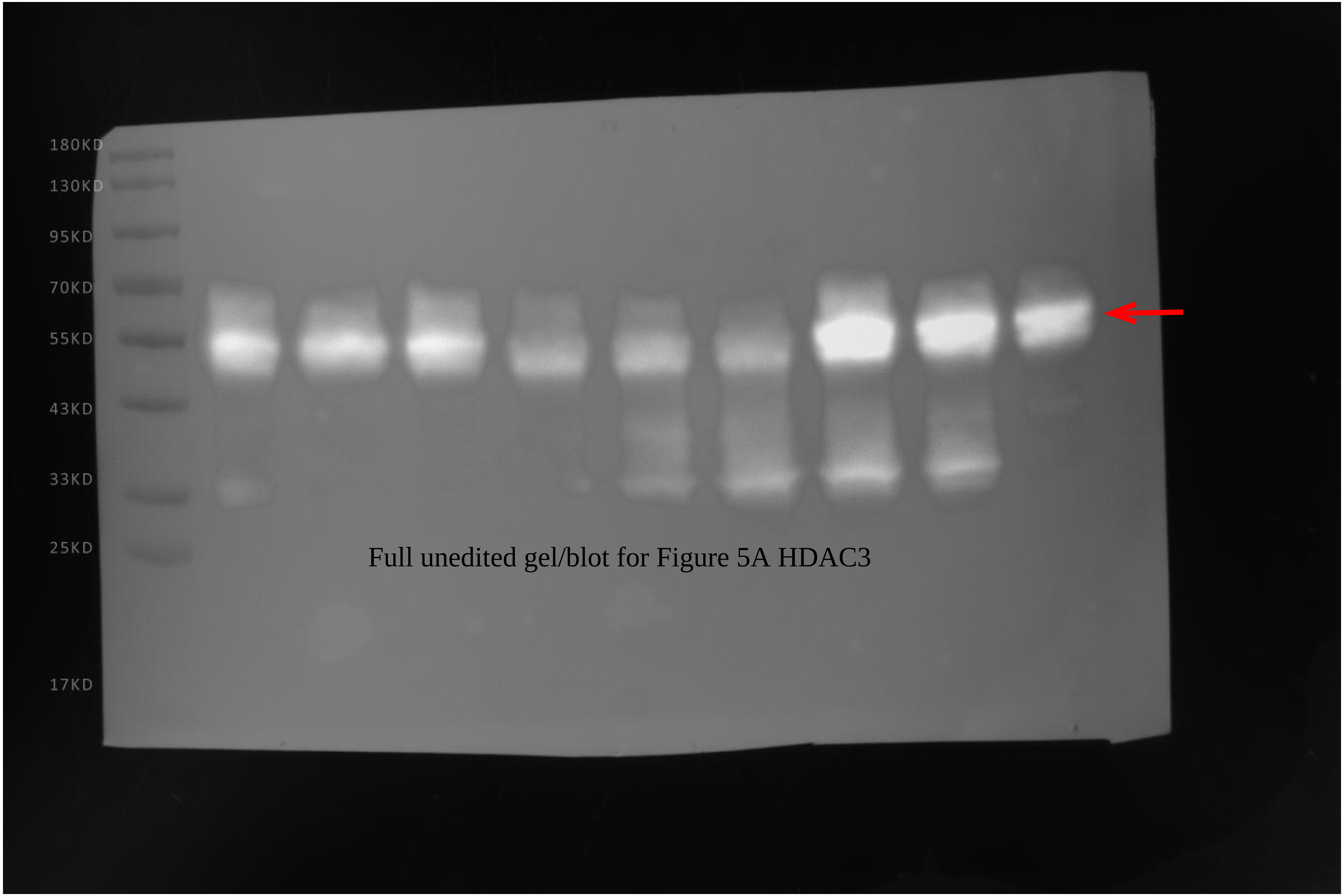

Full unedited gel/blot for Figure 5A HDAC3
