# Supplementary material for: Melatonin attenuates chronic sleep deprivation‐induced cognitive deficits and HDAC3‐Bmal1/clock interruption
Source: CNS Neurosci Ther. 2023 Sep 18;30(3):e14474. doi: 10.1111/cns.14474 (PMC10916425; doi:10.1111/cns.14474)
Supplement: Supplementary file 2 — Table S1. [file CNS-30-e14474-s001.docx]

Supplementary table 1: Primers used for real-time PCR

| Gene |  | Primer Sequence | Product Size (bp) |
| --- | --- | --- | --- |
| *Bmal1* | f  r | 5’-GTTTCAAATGTGGACCCCAAGG-3’  5’CAAAATCCTCCACCTAGACAGTCA-3’ | 198 |
| *Clock* | f  r | 5’-CCGAGACAGCTGCTGACAAA-3’  5’ -TCTTTGTCGGCGTTGAGGA-3’ | 177 |
| *Cry1* | f  r | 5’ -GGAAGAGGACGCACAGAG-3’  5’ -TCTCCCACCAACTTCAGC-3’ | 102 |
| *Cry2* | f  r | 5’- TAACCGTCAGCCCAAATC-3’  5’- AGGAGGGAGGAAGAAGAGT-3’ | 101 |
| *Per1* | f  r | 5’- GTCTTCTATGGCTCTACTACA-3’  5’- ACACGAATCTTGGTCACAT-3’ | 188 |
| *Per2* | f  r | 5’- AATCGTCCAACACTCACCC-3’  5’- CTCCGCAGAGCGTACTTC-3’ | 160 |
| *HDAC3* | f  r | 5’-AATGCCTTCAACGTGGGTGA-3’ 5’-CAGAAGCCAGAGGCCTCAAA-3’ | 179 |
